# PB.14: Visibility of screen-detected invasive carcinoma on digital breast tomosynthesis: do we need two views?

**DOI:** 10.1186/bcr3514

**Published:** 2013-11-08

**Authors:** O Abeyakoon, D Evans, R Rahim, J Morel, R Wasan, A Iqbal, J Golligher, C Peacock, M Michell

**Affiliations:** 1 Kings College Hospital, London, UK

## Introduction

Digital breast tomosynthesis (DBT) increases the sensitivity and specificity of detecting invasive breast carcinoma. Integration into screening raises questions. Should we perform two-view full-field digital mammography (FFDM) and two-view DBT or two-view FFDM and single-view DBT at every screening? DBT is shown to offer greatest benefit in the assessment of a soft tissue lesion. We routinely use two-view DBT in combination mode for all patients recalled from screening for a soft tissue abnormality. The aim of our study is to assess the need for two-view DBT in the detection of breast cancer.

## Methods

We retrospectively queried our histopathology database for all screen-detected invasive cancers between 2011 and 2012. The study sample was 254 cases. Comparisons were made between the visibility of the cancer on MLO/CC DBT and histological type/grade, molecular profile and breast density (BIRADS).

## Results

The mean age was 59 years. In total, 4/254 cancers were visible on one-view DBT (1.6%). (Two seen on MLO DBT [spiculated masses] and two seen only on the CC view [distortions]). A total of 11/254 had greater visibility on one view in comparison with the other view on DBT (4.3%). Two of 254 cases were occult on FFDM and DBT. Recall was for clinical symptoms, both lobular invasive carcinoma (0.79%). There was no relationship between histological type, grade or molecular characteristics and the visibility on one-view versus two-view DBT.

## Conclusion

A total 98.4% of cancers were seen on two-view FFDM and MLO-DBT. Integration of MLO-DBT into breast cancer screening with two-view FFDM is a consideration.

